# How much of the productivity losses among psoriasis patients are due to psoriasis

**DOI:** 10.1186/s12913-015-0752-0

**Published:** 2015-03-04

**Authors:** Anssi Mustonen, Kalle Mattila, Mauri Leino, Leena Koulu, Risto Tuominen

**Affiliations:** Department of Dermatology, Turku University Hospital and University of Turku, Turku, Finland; Department of Public Health, University of Turku and Primary Health Care Unit, Hospital District of Southwest Finland, Turku, Finland; Lemminkäisenkatu 1, 20014 University of Turku, Turku, Finland

**Keywords:** Absenteeism, Costs, Presenteeism, Psoriasis, Productivity

## Abstract

**Background:**

In previous studies, productivity losses have been measured specifically due to psoriasis or generally due to health problems in psoriasis patients. There is no information on the proportion of health related productivity losses that are due to psoriasis.

The aim of this study was to estimate the proportion of productivity losses due to psoriasis and due to other medical problems among employed psoriasis patients.

**Methods:**

Patients visiting a tertiary level dermatological clinic during a one-year period due to psoriasis or psoriasis arthritis, who were employed, were selected to the study. A questionnaire was used to assess productivity losses during the previous month.

**Results:**

Psoriasis accounted for 38% of the total lost productivity costs. One fifth of patients had been on sick leave (absenteeism) due to psoriasis and a third of patients worked despite being sick with psoriasis (presenteeism). Men had higher costs of presenteeism, but the costs of absenteeism due to psoriasis were lower for men than for women.

**Conclusions:**

Productivity losses should be assessed disease specifically to avoid overestimations of the role of the disease on indirect costs. Our study shows that about a third of the lost productivity costs are due to psoriasis.

## Background

Psoriasis is a chronic inflammatory disease, which generally affects people of working age [1]. Psoriasis reduces the ability of patients to work and may cause early retirement [2,3]. It has been estimated that patients with moderate to severe psoriasis suffer from a significant (15–20%) decrease in working ability [4-6].

The direct costs of treating psoriasis are substantial [7-10]. Indirect costs due to lost productivity have been estimated to exceed those of direct costs among patients with psoriasis and other chronic inflammatory diseases [11-15]. Other studies suggest that indirect costs are substantial but only contribute around 20–36% of the total costs [8,9,16-18].

Costs due to presenteeism are considered important when assessing the overall economic burden of psoriasis and other chronic diseases [1,17,19-22]. These costs are difficult to determine and were often omitted from studies in the past, although in the past decade, these costs have been increasingly included. However, the methods of measuring presenteeism are not consistent [1,15,20-22]. Presenteeism in psoriasis patients may account for around half of the indirect costs of lost productivity [8,14].

In previous studies [8,14,17,18,23] productivity losses have been measured specifically due to psoriasis or generally due to health problems. There is no information on the proportion of health related productivity losses that are due to psoriasis.

The aim of this study was to estimate the proportion of productivity losses due to psoriasis and due to other medical problems, and to assess the factors affecting presenteeism and absenteeism costs among employed psoriasis patients.

## Methods

### Patient sample

The sample was based on patients (498) who visited the Department of Dermatology in Turku University Hospital (TUH) between 1 October 2009 and 30 September 2010 with a diagnosis of psoriasis (Ps) or psoriatic arthritis (PsA). In the Finnish healthcare system, patients with mild psoriasis are treated in primary health care settings and only moderate to severe cases are referred to tertiary level hospitals for further treatment. In practice, all psoriatic arthritis patients in this study sample also had skin symptoms, which had been the reason for visiting a dermatological clinic. These patients were asked to complete a questionnaire. A total of 262 patients completed the questionnaire (52.6% of the total study sample). The share of patients with PsA diagnosis was 13% in all patients (498) visiting TUH and those who completed the questionnaire (262).

### Ethical consideration

The ethical committee of The Hospital District of Southwest Finland approved the study. The patients received a written description of the sampling procedure and study purpose, as well as the planned use and storage of the information they were to provide. This was followed by a description of the subject’s rights according to the Helsinki declaration. The patients gave a written consent to use their medical records for the study.

### Questionnaire

Socio-demographic background information (e.g. sex, age, home municipality, number of people living in the same household, gross income level per family member), and disease duration were collected. Patients were asked to list all possible concomitant diseases. Due to small number of various concomitant diseases, a dichotomy was formed as: 0 = no other illnesses, 1 = having at least one concomitant disease. Subjects were asked to report whether they were employed, retired, studying or unemployed, with multiple choices allowed. Of the sample, 98 patients reported that they were working during the study period, and comprised the sample used for this study.

Absenteeism was assessed by asking: “How many hours during the past 4 weeks have you been away from work due to psoriasis?” A similar question followed to assess absenteeism arising from other medical reasons.

Presenteeism was assessed by asking: “How many hours during the past 4 weeks have you been working while sick, when you felt that you should have stayed at home because of your psoriasis?” To quantify the loss of productivity during the hours a patient worked while sick, a 100 mm visual analogue scale (VAS) was used, with 0 representing not at all affected and 100 representing affected extremely much, with the following question: “Please mark an X on the line, to describe the decrease in efficacy at work because of your psoriasis, during the hours in the last 4 weeks that you worked while sick?” Similar questions were used to determine the amount and impact of presenteeism due to other medical reasons.

### Time costs

All time estimates were computed to hours per year. The VAS score (mm) of lost productivity for presenteeism was divided by 100 to indicate the magnitude of lost productivity during the hours the patient worked while sick. This was used to multiply the hours per year to give an estimate of productivity loss due to presenteeism.

To estimate the monetary value of the productivity loss a time-cost assessment was used. The value of an hour was estimated using the Human Capital Approach (HCA). The value was based on the average monthly income in Finland of €2807 for women and €3422 for men (Statistics Finland 2011). The monthly income levels were computed to an hour based on average working hours (157 hours per month) in Finland for people working full-time. The gross income per family member, asked in the questionnaire, was not used for time cost computations as it could have resulted in underestimation of costs for patients with many family members. It was only used in the analyses as a background factor indicating purchasing power.

### Statistical analyses

The statistical evaluation of the data was based on Student’s *t*-test for means and chi-square test for proportions. Patients with missing data were not included in respective cost estimations and statistical analyses. Linear and logistic regression models were used to study the impact of different background factors on the estimates of lost productivity costs. In the case of skewed distribution to the left (as in the total costs of absenteeism, presenteeism and total productivity losses), natural logarithmic transformation was used to obtain close to normal distribution, which was necessary for linear modelling. Logistic and linear regression models of the productivity costs were studied with the following background factors: sex (dichotomy: 0 = women, 1 = men), disease duration (in years), concomitant diseases (dichotomy: 0 = no other illnesses, 1 = having at least one concomitant disease) and level of income (per family member). There were no statistically significant differences between patients with skin symptoms only and patients with skin symptoms and arthritis in any of the analyses made. Thus, these two patient groups were analysed as one psoriasis group. The use of different treatments, disease severity and quality of life were similar and without statistical significance between men and women. Thus, these background factors were not included in final statistical models.

## Results

The employed (n = 98) patients of the study sample were on average 49 years old, 55% were male and 13% were diagnosed to have PsA. During the last 4 weeks, approximately one fifth of employed patients (19%) had been on sick leave (absenteeism) due to psoriasis and 28% of patients reported that they had worked despite being sick with psoriasis (presenteeism). Psoriasis accounted for 38% of the total costs due to lost productivity (Table 1).

**Table 1:** **Annual mean costs (€) of absenteeism and presenteeism by sex**

	**Total**	**Men**	**Women**	**p-values**
Absenteeism due to **psoriasis**	1105 (5045)	355 (987)	2032 (7419)	(p = 0,151)
Presenteeism due to **psoriasis**	1037 (2776)	1453 (3441)	535 (1566)	(p = 0,123)
Productivity loss due to **psoriasis**	2250 (6247)	1857 (3943)	2728 (8283)	(p = 0,563)
Absenteeism due to other medical reasons	2494 (6832)	2858 (8165)	2045 (4784)	(p = 0,609)
Presenteeism due to other medical reasons	1560 (4036)	1882(4476)	1169 (3446)	(p = 0,430)
Productivity loss due to other medical reasons	4172 (8807)	4765 (9702)	3417 (7597)	(p = 0,514)
Absenteeism **Total** (Due to psoriasis and other medical reasons)	3200 (7944)	2531 (7478)	4011 (8520)	(p = 0,432)
Presenteeism **Total** (Due to psoriasis and other medical reasons)	2307 (5941)	2829 (7212)	1638 (3748)	(p = 0,365)
**Total productivity loss** (Due to psoriasis and other medical reasons)	5409 (10515)	4800 (10272)	6160 (10935)	(p = 0,603)

Footnote: Subjects with missing data in any category were excluded from respective cost estimations. Thus, total loss estimates may differ from summing up costs.

Productivity loss costs displayed separately due to psoriasis, due to other medical reasons and the total productivity loss costs (Standard deviation in parenthesis), p-value representing statistical significance between men and women.

Absenteeism due to other medical reasons was 2.5 times more common than absenteeism due to psoriasis (Table 1). For the patients who reported absenteeism, the estimated mean annual work time lost due to psoriasis was 306 hours, corresponding to a mean cost of absenteeism of €6296 per year and a median cost of €2092 per year.

The estimated presenteeism costs due to psoriasis were around 50% lower than for presenteeism due to other medical reasons (Table 1). For the patients who reported presenteeism due to psoriasis, the estimated mean annual duration was 391 hours. During these hours, the decrease in productivity was on average 45% (range 8–85%), which led to a mean cost estimate of €3605 per year and a median cost of €1647 for presenteeism.

Men and women had similar basal characteristics with no statistically significant differences in socio-demographic background factors, disease severity, quality of life or treatment strategy. Men worked while sick due to psoriasis for a longer period of time and had a greater decrease of productivity than women and thus, higher costs of presenteeism. However, the costs of absenteeism due to psoriasis were lower for men than for women (Table 1). Costs of absenteeism and presenteeism due to other medical reasons were higher for men than women (Table 1).

In logistic regression models the background variables studied did not show any significant effect on the likelihood of a patient reporting absenteeism, presenteeism, or any productivity loss due to psoriasis. The explanatory effect of the background variables was very limited, with Negelkerke R values at 0.07 level in all these models.

In a linear regression model, absenteeism costs due to psoriasis were significantly higher for women than men (Table 2). The only statistically significant background factor affecting presenteeism costs due to psoriasis was having concomitant diseases (Table 2). Other background factors did not have a statistically significant effect on absenteeism or presenteeism costs due to psoriasis (Table 2).

**Table 2:** **Linear regression models for natural logarithmic transformations (ln 1 + costs) of absenteeism and presenteeism costs due to psoriasis**

	**Absenteeism**			**Presenteeism**		
	Beta	t-stat	p=	Beta	t-stat	p=
Male sex	-0,609	-2,359	0,040	0,398	1,402	0,180
Income level	-0,322	1,243	0,242	-0,410	-1,553	0,140
Disease duration	0,199	0,859	0,569	0,375	1,687	0,111
Concomitant diseases	0,393	1,095	0,299	0,446	2,208	0,042
	R^2^ = 0,4			R^2^ = 0,38		

The linear regression models were subjected to a sensitivity analysis, in which women’s income level was gradually increased up to 20% higher. The models showed that observed differences between sexes were not sensitive to women’s salary level.

## Discussion

The findings from the present study indicate that psoriasis is not necessarily the major health related reason causing productivity losses among patients with psoriasis. In this relatively small sample, other medical reasons produced two-thirds of the overall productivity losses. In two separate studies in the US, Schmitt and Ford [14] estimated that the indirect costs of psoriasis were higher than the estimated direct costs [7]. In the study by Schmitt and Ford [14] the productivity losses were estimated at a general level (that is, assessing productivity losses due to any health problems), which may have led to overestimating the role of psoriasis in the productivity loss costs. In the present study, the costs per patient of lost productivity due to psoriasis were half of those estimated by Schmitt and Ford [14]. The costs of productivity loss due to any medical reason (including psoriasis) were higher than the costs estimated by Schmitt and Ford [14]. In several studies [8,9,24] the costs of lost productivity were only those due to or related to psoriasis. The proportions of direct and indirect costs of lost productivity may be shifting towards direct costs, as more expensive biologic medications are being increasingly used.

Male patients with psoriasis may consider that psoriasis is an illness that does not require absenteeism from work, whereas women may more readily take sick leave. This conclusion is supported by the findings that male patients with psoriasis had higher costs of absenteeism due to other illnesses and higher costs of presenteeism than women with psoriasis. To our knowledge, there are no previously reported or observed differences between genders in productivity losses due to psoriasis. Studies on other diseases have estimated that presenteeism is more common for women [25,26] and there are mixed results for absenteeism [25,27]. The differences between genders in psoriasis patients may be explained by different attitudes towards visible lesions or other cosmetic hindrance. The reluctance of male psoriasis patients to take sick leave when ill may lead to increased severity and duration of psoriasis, leading to increased presenteeism. However, further studies with larger samples are needed to corroborate our findings.

Concomitant diseases have a significant effect on the overall wellbeing of patients, and a significant effect on the costs of lost productivity for patients with psoriasis [18] and other chronic diseases [12]. In our study, concomitant diseases significantly increased presenteeism costs, but not absenteeism or total productivity costs due to psoriasis. However, the small number of various concomitant diseases among the study patients did not allow specific comparisons on which type of concomitant diseases had the greatest effect on productivity loss costs.

It has been stated that there are 16 popularly used instruments to estimate presenteeism, hampering comparisons between studies using different measurements [20]. In a study where different measures for presenteeism were evaluated, there was a four-fold difference in cost estimates between the measures providing the highest and the lowest cost estimations [28]. A recent study on rheumatoid disorders found inter-country variation in presenteeism costs when the same measures and methods are used, suggesting that findings are not directly transferable from country to country [27]. Even though, given the inconsistencies between methods of previous studies, our study’s results on the proportion of psoriasis of productivity loss may be somewhat generalizable to other psoriasis patients regardless of methods used. However, in general questions of absenteeism, and in particular of presenteeism may be difficult for the patients to interpret. This can be pronounced when the questions are dealing with disease as a whole, instead of specific symptoms (e.g. itch, arthralgia). This may be a possible source of bias. On the other hand, in our opinion, patients should not have difficulties in distinguishing between “due to psoriasis” and “due to other health related problems”.

The costs of early retirement or unemployment can be substantial; they were estimated to form 92% of lost productivity costs due to psoriasis [11]. However, retirement or unemployment are rarely due only to psoriasis or other chronic diseases and are likely to be affected by concomitant diseases and other health problems. Our study did not assess the reasons for retirement or unemployment. Consequently, any cost estimations of retirement or unemployment could have resulted in unreliable estimations and probable overestimation of these costs, and thus they were not included in this study.

Caution is advised when of the costs of lost productivity are extrapolated into economic burden (20). The HCA method has been criticized for overestimating the costs, particularly over time horizons greater than 1 year, as in early retirement [20]. Furthermore, HCA may not consider all possible costs that the absence of a worker causes to an employer, and omits the costs of unemployed and retired patients [29]. In this study the aim was to estimate the productivity loss of employed patients, thus omitting the retired and unemployed was considered valid. The present study focused on a societal perspective of the productivity losses, thus the HCA method was used. The losses to employers may be significantly higher, as costs of reduced productivity may exceed the salaries of employees. It has been estimated that losses to employers can be up to three times the gross salaries of sick listed employees [30].

There is no consensus on the time frames that should be used in questionnaires to assess productivity loss. A 2-week timeframe has been suggested for presenteeism cost estimations to minimize recall bias of patients and improve generalizability [20]. However, a recent review stated that the scientific research on presenteeism and absenteeism recall periods is inconclusive, but recommended a 3-month recall period for absenteeism and a 1-week recall period for presenteeism [29]. However, the rationale for using different time frames for absenteeism and presenteeism has been questioned [22].

The costs of lost production can be minimized with efficient treatment of autoimmune diseases, as patients with few symptoms or low severity perform on a par with healthy co-workers [19]. It has been suggested that the high acquisition costs of biological treatments could be offset by improved work productivity and decreased need for inpatient and outpatient visits [12,31-33]. However, productivity losses should be assessed specifically for the disease to avoid overestimations of the role of the disease on indirect costs of lost productivity. Our study shows that, for patients with psoriasis treated in a tertiary clinic, a third of the lost productivity costs are due to psoriasis. More studies with larger sample sizes and from other societies are needed to verify the proportion that psoriasis contributes to overall productivity losses.

## Conclusion

Productivity losses are significant in patients with psoriasis. However, only a third of the productivity losses of psoriasis patients are due to psoriasis.
